# Analysis of whole exome sequencing in severe mental illness hints at selection of brain development and immune related genes

**DOI:** 10.1038/s41598-021-00123-x

**Published:** 2021-10-26

**Authors:** Jayant Mahadevan, Ajai Kumar Pathak, Alekhya Vemula, Ravi Kumar Nadella, Biju Viswanath, Sanjeev Jain, Accelerator Program for Discovery in Brain disorders using Stem cells (ADBS) Consortium, Naren P. Rao, Janardhanan C. Narayanaswamy, Biju Viswanath, Palanimuthu T. Sivakumar, Arun Kandasamy, Muralidharan Kesavan, Urvakhsh Meherwan Mehta, Ganesan Venkatasubramanian, John P. John, Meera Purushottam, Odity Mukherjee, Ramakrishnan Kannan, Bhupesh Mehta, Thennarasu Kandavel, B. Binukumar, Jitender Saini, Deepak Jayarajan, A. Shyamsundar, Sydney Moirangthem, K. G. Vijay Kumar, Bharath Holla, Jayant Mahadevan, Jagadisha Thirthalli, Prabha S. Chandra, Bangalore N. Gangadhar, Pratima Murthy, Mitradas M. Panicker, Upinder S. Bhalla, Sumantra Chattarji, Vivek Benegal, Mathew Varghese, Janardhan Y. C. Reddy, Sanjeev Jain, Padinjat Raghu, Mahendra Rao, Meera Purushottam, Mayukh Mondal

**Affiliations:** 1 Department of Psychiatry, National Institute of Mental Health and Neurosciences, Bangalore, India; 2 Institute of Genomics, University of Tartu, Tartu, Estonia; 3 National Institute of Mental Health and Neurosciences, Bangalore, India; 4 Institute for Stem Cell Biology and Regenerative Medicine (InStem), Bangalore, India; 5 National Center for Biological Sciences (NCBS), Bangalore, India

**Keywords:** Genetics, Behavioural genetics, Evolutionary biology

## Abstract

Evolutionary trends may underlie some aspects of the risk for common, non-communicable disorders, including psychiatric disease. We analyzed whole exome sequencing data from 80 unique individuals from India coming from families with two or more individuals with severe mental illness. We used Population Branch Statistics (PBS) to identify variants and genes under positive selection and identified 74 genes as candidates for positive selection. Of these, 20 were previously associated with Schizophrenia, Alzheimer’s disease and cognitive abilities in genome wide association studies. We then checked whether any of these 74 genes were involved in common biological pathways or related to specific cellular or molecular functions. We found that immune related pathways and functions related to innate immunity such as antigen binding were over-represented. We also evaluated for the presence of Neanderthal introgressed segments in these genes and found Neanderthal introgression in a single gene out of the 74 candidate genes. However, the introgression pattern indicates the region is unlikely to be the source for selection. Our findings hint at how selection pressures in individuals from families with a history of severe mental illness may diverge from the general population. Further, it also provides insights into the genetic architecture of severe mental illness, such as schizophrenia and its link to immune factors.

## Introduction

Severe mental illnesses (SMI) such as schizophrenia and bipolar disorder (BD) have a lifetime prevalence of 1%; and this seems to have remained relatively stable across geography and time^1,2^. Psychiatric disease syndromes are common, usually begin in young adulthood, are a source of considerable personal and social distress, associated with premature mortality and the treatments have limited efficacy. Hence, detecting underlying mechanisms that may contribute to risk and recovery will be useful.

These syndromes are known to be heritable and their genetic architecture is quite likely to be polygenic, with a combination of common variants of small effect and rare variants of relatively larger effect being implicated^3^. The apparently uniform genetic epidemiology of these syndromes in different parts of the world seems to suggest that there is no specific selection for, or against, these conditions. This has been attributed to theories of balancing selection, ancestral neutrality or polygenic mutation selection balance^4^.

From a population genetics standpoint, admixture, migrations and selection all have an impact on our understanding of the genetic contributions to risk of psychiatric illness^5^. Summary statistics generated from genome wide association study (GWAS) data have been commonly used to investigate the contribution of natural selection on the genetic architecture of complex traits, such as psychiatric syndromes^6^. Findings from studies investigating the role of natural selection in mental illness have been ambiguous with a few implicating the role of positive selection^7–9^, while others have shown either no evidence for selection or negative selection^4,10,11^.

Whole exome sequencing, which documents the variation in protein-coding sequences, has also been used as a tool to investigate natural selection. A number of studies in isolated populations have identified genetic variation that confers protection against environmental conditions such as adaptation to hypoxia at high altitudes among Tibetans^12^ or arctic climate among Nunavik Inuit^13^ and Siberians^14^. Signatures of natural selection are detected even in the context of more recent population divergence and this influences many aspects of physiology, underscored by variations in genes that impact on height, blood coagulation, pigmentation, diet availability and resistance to infections^15,16^.

People with psychiatric illness such as schizophrenia and BD are known to have protective alleles in their genomes^17^, and these may be associated with resilience^18^. A study which investigated the evolutionary pattern in the SLC39A8 gene, found that a schizophrenia risk variant in the European population had experienced recent positive selection in Europeans, and that it may have offered protection from the risk of hypertension, and also helped them adapt to the cold environment^19^. These patterns have been suggested, and detected, for many diseases, especially those that have an onset in adult life^20^.

In addition to the role of natural selection, there has also been growing interest in understanding the contribution of archaic sequences of DNA to liability for disease^21^. We know that there has been more than one instance of admixture between early human populations along with Neanderthals and Denisovans^22,23^. This has resulted in the persistence of a number of introgressed sequences of archaic (Neanderthal and Denisovan) DNA that account for around 2–4% of the genome in modern (*Homo sapiens*) human populations. Studies have demonstrated the influence of such sequences on immune functioning and susceptibility to infections including COVID-19^24^. These sequences have also been found to be depleted in genes related to specific brain regions^25^ and influence functional connectivity in the brain as well^26^. Consequently, the impact of archaic introgression and admixtures on psychiatric disorders merits further exploration. South Asia has been inhabited by modern humans for the last several thousand years, and the population displays admixture with both extinct hominins, as well as significant migrations and bottlenecks in the recent past^27,28^. These admixture events may thus have a noteworthy influence on the susceptibility and prevalence of disease.

Hence, in this study, we investigated signatures of positive selection in unrelated individuals from families with multiple affected individuals with severe mental illness from southern India. We also used allele frequency differences between the cases and controls from the same population to prune out potential regions directly associated with caseness, and concentrated on regions with strong positive selection. Additionally, we specifically explored whether genes which showed evidence of positive selection had any evidence of Neanderthal introgression.

## Results

We used whole exome sequence (WES) data of 80 unrelated individuals each of whom was diagnosed with psychiatric illness, as cases. These individuals were drawn from 80 separate and distinct families in which multiple members (at least 2 first-degree relatives in a nuclear family) were diagnosed to have a major psychiatric disorder [schizophrenia, BD, obsessive compulsive disorder (OCD), dementia and substance use disorders (SUD)]^29^ (A description of the sample is provided in the “Methods” section).

Since WES data is highly dispersed, we decided to use Population Branch Statistics (PBS) for our selection analysis^30^. PBS is based on allele frequency differentiation between populations using three populations. Unlike F_ST_^31^, PBS is directional and gives us a clear idea as to which population is under selection for the given allele. A high PBS value corresponds to a highly deviated allele frequency of the target population compared to the reference population caused by positive selection.

Here, we used our data set consisting of 80 cases as the target population, the South Asian and African ancestry genomes from the gnomAD dataset as reference and outgroup populations respectively^32^. We also used WES data from 10 unrelated individuals from the same population as controls in the analysis to exclude PBS differences that may be attributable to case status rather than selection (A description of the same is provided in the “Methods” section).

Further, since the gnomAD dataset only reports South Asian ancestry Samples (SAS), which is a super population, we also tried to test if using a super population may bias our results when using the same as a reference population for the PBS analysis. We used 1000 genome 3rd phase data, which provides labels of South Asian subpopulations [such as Indian Telugus from the United Kingdom (ITU) and Gujarati Indian from Houston (GHU)], for this purpose. We found that PBS values coming from a subpopulation and superpopulation were highly correlated (R^2^ = 0.8534), especially SNPs with top values were common between both the results (Fig. S1). This reiterates our previous results^33^, and supports the conclusion that the use of the SAS super population in our study did not influence the findings of the PBS analysis.

### Identification of SNPs and genes under influence of selection

We followed an approach that defined the SNPs that fell in the top 0.1% (99.9th percentile) of the PBS value distribution as the most likely candidates for selection. Further, to increase the confidence that the SNPs that fell under extreme PBS values were caused by selection rather than sampling of cases, we calculated the frequency difference of these SNPs between cases and controls. We then excluded all SNPs (Table S9) that fell within the top 0.1% (99.9th percentile) of the frequency difference distribution between cases and controls, also supported by the Fisher’s exact test P value and Odds ratio (OR) for frequency difference in cases and controls. This provided a list of candidate genes which were a plausible target of adaptive evolution specific to our test population. Further, we filtered genes with at least two SNPs with high PBS value to reduce the chance of false positives.

**Figure 1:**
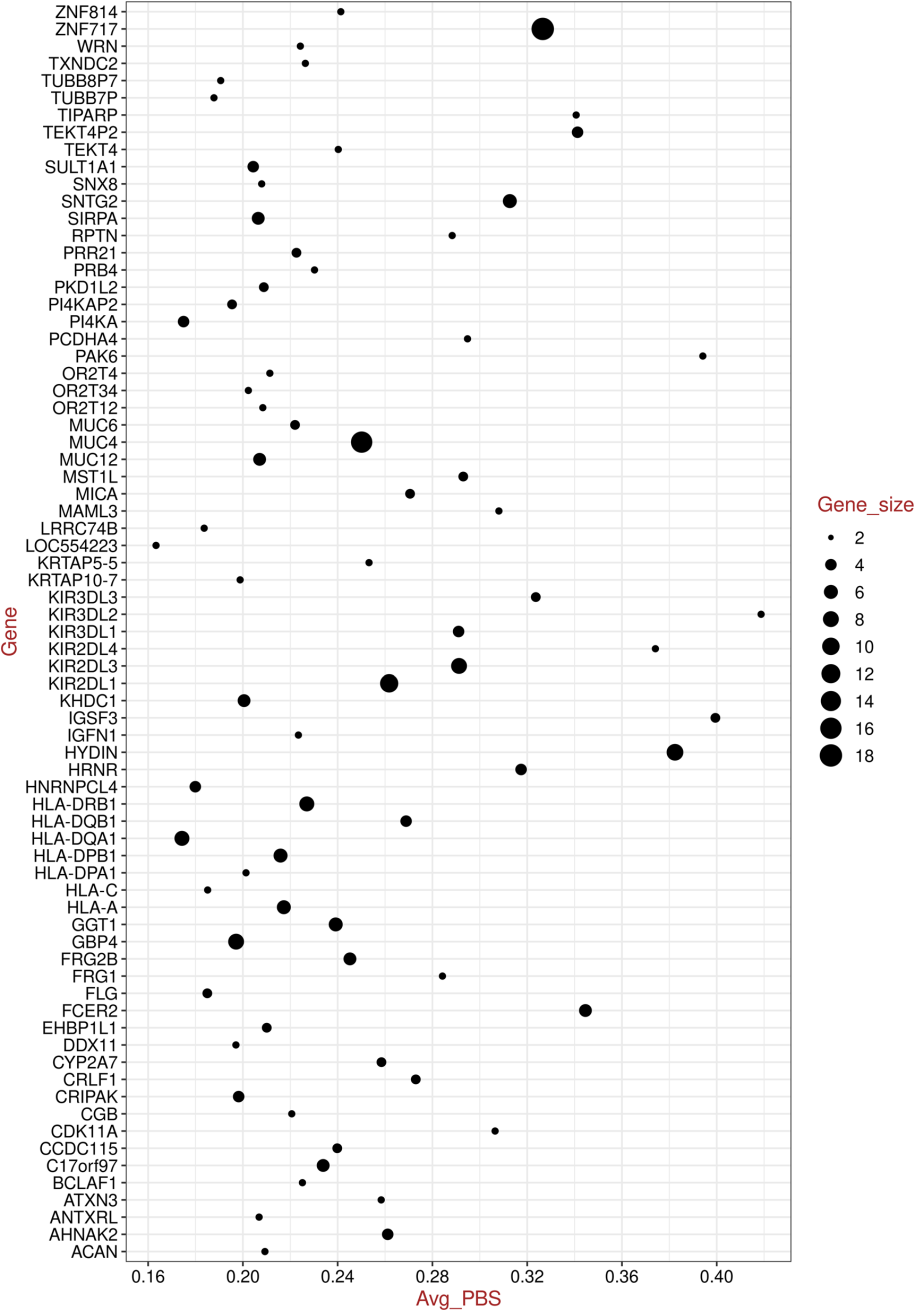
Dot plot of genes with multiple SNPs against the average PBS value; the dot size varies based on gene size (number of SNPs). While plotting we removed the gene *SARM1* that was behaving as an outlier (average PBS value = 0.89591), for better visualization.

A total of 398 SNPs located in 190 genes were found to lie in the top 0.1% of the PBS value distribution. 115 genes had one SNP per gene (Table S1), while 75 genes had more than one SNP per gene (Table S2). A total of 10 SNPs from 10 genes were excluded due to case - control differences (Table S9). After this, we had 110 genes that had only one SNP per gene (Table S3) and 74 genes that had more than one SNP per gene (Table S3). For these 74 genes, we calculated an average PBS value per gene (Fig. 1; Table S3). We then used this list of 74 genes to look for any indications of an underlying shared biology.

### Functions of putatively selected genes

Many of the 74 genes are involved with immunological and defense responses including activation and regulation of interferon-gamma, cytokine and immune system, and different signaling pathways. We manually curated these genes using the GWAS Catalogue (https://www.ebi.ac.uk/gwas/) and ENSEMBL (https://www.ensembl.org/Homo_sapiens/Info/Index) and found that several of the listed genes were reported for having potential roles in cancer, liver disease and diabetes.

We found 20 genes (*MAML3, SNX8, WRN, ATXN3, PAK6, TXNDC2, MICA, PK1LD2, AHNAK2, PI4KA, C17orf97, FCER2, SNTG2, GGT1, FLG, IGFN1, PCDHA4, ANTXRL SULT1A1*), in this list of 74 genes, that have been previously associated with elevated risk for schizophrenia, Parkinson disease, Alzheimer’s Disease and cognitive abilities or intelligence (Table S4).

### Functional enrichment and pathway enrichment analysis

Furthermore, to evaluate whether genes with extremely high PBS values (the 99.9th percentile) were enriched in any functional category or metabolic pathways, we evaluated our list of 74 genes for the three Gene Ontology (GO) categories: biological processes (Table S5), cellular components (Table S6), and molecular function (Table S7). In addition, we analyzed the gene list for pathway over-representation using the IMPaLa tool (Table S8).

In GO analysis, we observed that several of selected genes of the target population were functionally enriched (P < 0.05) for different signaling and regulatory mechanisms related to immune system, and viral defense such as negative regulation of natural killer cell mediated cytotoxicity, interferon-gamma-mediated signaling pathway and antigen processing and presentation of exogenous peptide or polysaccharide antigen via MHC class II.

Using the IMPaLa pathway analysis tool, we again observed an over-representation for the enrichment of genes (below Q-value 0.05) involved in different immune related pathways including antigen processing and presentation, Graft-versus-host disease, Type I diabetes mellitus and autoimmune thyroid disease.

We repeated the functional enrichment analysis and pathway analysis with our list of 74 genes after exclusion of the HLA region (which is known to be extremely polymorphic). It was seen that while the GO analysis did not show any evidence of functional enrichment, the pathway analysis using IMPaLA suggested pathways involved with immune function (Table 1).

**Table 1:** IMPaLa pathways enrichment analysis results for the list of 74 multiSNP genes after removing HLA genes.

Pathway_name	Pathway_source	Num_overlapping_genes	Overlapping_genes	Num_all_pathway_genes	P_genes	Q_genes
Antigen processing and presentation—*Homo sapiens* (human)	KEGG	6	KIR3DL1; KIR3DL2; KIR3DL3; KIR2DL1; KIR2DL3; KIR2DL4	77 (77)	8.44E−-08	0.000387
Natural killer cell mediated cytotoxicity—*Homo sapiens* (human)	KEGG	6	KIR3DL1; KIR3DL2; MICA; KIR2DL1; KIR2DL3; KIR2DL4	130 (131)	1.90E−06	0.00435
Graft-versus-host disease—*Homo sapiens* (human)	KEGG	4	KIR2DL3; KIR2DL1; KIR3DL1; KIR3DL2	41 (41)	5.83E−06	0.00892
Immunoregulatory interactions between a Lymphoid and a non-Lymphoid cell	Reactome	6	KIR3DL1; KIR3DL2; MICA; KIR2DL1; KIR2DL3; KIR2DL4	218 (221)	3.67E−05	0.0421
Termination of O-glycan biosynthesis	Reactome	3	MUC4; MUC6; MUC12	26 (26)	5.73E−05	0.0526

### Archaic introgression in putatively selected genes

**Figure 2:**
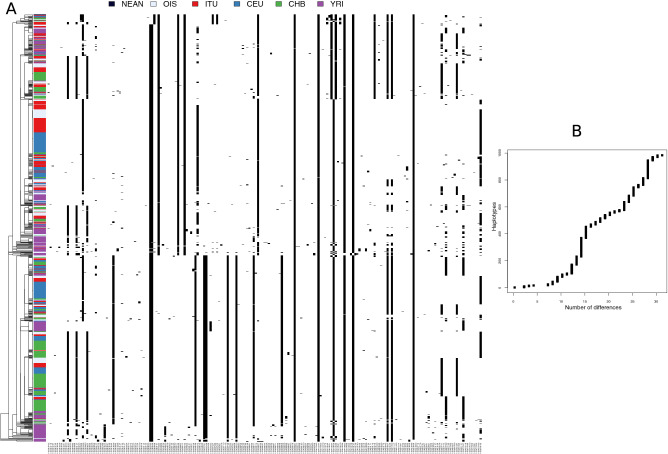
Haplostrips plot of *AHNAK2* gene: (**A**) Clustered and sorted by increasing distance with Neanderthals. A few Neanderthal derived SNPs show a pattern of unique haplotype sharing among the continental populations. However, none of these SNPs were found in high PBS value during selection scan and therefore have no significance in the adaptive role of *AHNAK2* in this study. Population label abbreviations are as follows: *NEAN* Neanderthal, *OIS* Our Indian Samples, *ITU* Indian Telugus, *CEU* Central Europeans from Utah, *CHB* Chinese Han from Beijing, *YRI* Yoruba. (**B**) This plot visualizes the extent of closeness (based on SNP difference) between the haplotypes shared by continental populations and Neanderthal.

Further, we looked for the archaic introgression in these 74 genes that were chosen as candidates of selection. We applied Haplostrips^34^ to the exome data of our 80 unrelated case samples. However, we could not detect any definitive traces of archaic introgression in any of the genes in the gene sets except *AHNAK2* gene (Fig. 2). Though we observed a few Neanderthal derived SNPs in the *AHNAK2* gene region showing a pattern of unique haplotype sharing among the continental populations, none of these SNPs were found in high PBS value during selection scan.

The fact that Neanderthal introgression sequence in the *AHNAK2* gene region does not contain any of the variants identified as putative candidates of selection, indicates that the introgressed haplotype of the gene was probably not selected, thus, rules out its significance in the adaptive role of AHNAK2 in this study.

## Discussion

Our results identify a total of 74 genes that show definitive evidence of selection, in individuals coming from families with multiple individuals with severe mental illness from southern India, based on the PBS analysis. Many of the identified genes have complex biology with numerous being linked to immune processes, cancer and neuropsychiatric illnesses.

There is some prior suggestion that many genes implicated in brain function, and disease, may be under selection^35^. This was supported by our findings where genes that we identified to be under selection have been previously implicated in a number of neuropsychiatric disease phenotypes. These include; *SNX8* and *PAK6*, which were seen to harbour common variants that were associated with schizophrenia in GWAS^10,36–38^, *SNTG2* and *PKD1L2* that were associated with neurodevelopmental disorders such as autism^39^ and attention deficit hyperactivity disorder^40^, and *ATXN3* and *C17orf97* that have variants associated with neurodegenerative disorders like Amyotrophic Lateral Sclerosis^41^ and Parkinson's disease^42^. We also found genes related to disorders of neuronal migration and brain malformations such as polymicrogyria, like *AHNAK2* and *PI4KA*^43^.

Aside from associations with disease phenotypes, a number of the genes we identified have been implicated in brain biology, including neurodevelopment, maintenance of neuronal and axonal integrity and apoptosis. The SARM1 protein is a member of MyD88/TIR-domain containing superfamily of proteins, which are involved in innate immune responses. Deficiency of the protein is seen to influence the apoptotic cascade of a variety of neural cells, including microglia; and cytokine expression in the brain^44^. The HYDIN protein, associated with cilia in the brain, is critical for development of ventricles and brain; and it interacts with other genes like *FOXP2* which are well known to be related to neurodevelopmental disorders such as ASD and ADHD^45^. The neurodevelopmental disorders of autism and schizophrenia have also been hypothesised to be linked to UV exposure possibly via vitamin D levels^46^. Thus, it was interesting to identify the *HORNERIN / HRNR* gene which is very sensitive to UV light and is believed to also be involved in the species-specific differentiation of the outer layer of skin^47^. Further, genes identified by us such as *MAML3*, *WRN* and *SULT1A1* also harbour variants linked to intelligence and cognitive abilities^48–50^. The contributions of these genes to neurodevelopment, intelligence and cognitive abilities suggests why they may be plausible candidates undergoing positive selection.

We also identified a number of genes linked to immune functions that were under selection in our sample. This was reflected in our functional enrichment and pathway enrichment analyses, where biological processes and pathways linked to the immune system were strongly implicated. This is expected, since immune function related genes are known to undergo significant selection pressure by virtue of factors such as the need to adapt to ecological diversity, and biological factors such as the threat posed by new and ever-changing infectious pathogens^51,52^.

The concurrent selection of genes that influence distinct, and apparently disparate biological processes, raises a number of extremely interesting questions about the interplay between the immune system and neuropsychiatric phenotypes. A well-known example is the role of the *ApoE ε4* allele, which confers protection against viral illness on the one hand, but increases the risk of cognitive impairment later in life^53^. The role of the HLA region on Chromosome 6 has also been a consistently reported finding from GWAS and WGS of schizophrenia^54^. A study which investigated the role of balancing selection using exome data from modern and archaic humans also found an excess of SNPs across species in a gene set associated with the immune system, of which six were located within genes previously associated with schizophrenia^55^. Another study also suggests shared genetic pathways linking white blood cell indices and complex diseases such as schizophrenia, autoimmune, and coronary heart diseases^56^.

A number of genes that we identified in our study, such as the KIR family of genes have been implicated in a number of immune related functions, and also influence neurodevelopment and immune reactions in the brain. The *KIR3DL1* has been linked to several aspects of natural killer cell responses, which in turn have been linked to susceptibility to multiple sclerosis, especially in African-Americans^57^. The KIR2DL4 immunoglobulin-like receptor has also been implicated in the development and maintenance of oligodendrocytes^58^; and also thought to have been positively selected for enabling uterine tolerance for embryonic implantation in humans^59^. Similarly, the *KIR3DL2* have also been implicated in the interface between immunity genes and brain development, inflammation and responses to damage. They are the receptors for the major histocompatibility complex class I (MHC-I) like HLA-F, and protect neurons from astrocyte induced toxicity, as is seen in ALS^60^. The *IGSF3* gene forms a complex with Tetraspanin (*TSPAN7)*, which is involved in neurodevelopment (many mutations in this gene are linked to X-linked syndromes), and thus mediates a cross-talk between immune mechanisms and development^61^.

Although we observed archaic introgression in a single gene (*AHNAK2*) out of the identified set of putative selected genes, we did not find any evidence of archaic introgression under positive selection in any of the genes that were identified using the PBS analysis. These could be related to the fact that exome sequences have a restricted utility when it comes to finding introgressed regions, as the exonic regions are short and spaced, in the context of the whole genome. Additionally, from an evolutionary standpoint, selection tends to happen either upstream or downstream of the genes in areas such as transcription binding sites, rather than in the exonic regions which are well conserved^62^. Furthermore, exonic regions are generally under negative selection and therefore may not exhibit differences between modern and archaic hominins. Therefore, even if some highly differentiated exonic segment introgressed, it is most likely to have been weeded out by negative selection from the population, as Neanderthals have many more deleterious SNPs due to their low effective population size^63^. Thus, while it is not impossible to find an introgressed exonic region being selected for, these are rare in the literature^64^.

A few previous studies looking for selection signatures in south Asian populations using different methodologies have found evidence for positive selection in genes related to lipid metabolism and glucose uptake and have posited a link between the same and the predilection towards development of type 2 diabetes and obesity^65^ and height in Andaman Island populations^66^. Hence, our study also provides fresh insights into selection in a population from southern India.

In conclusion, our findings show that families with multiple members affected with severe mental illness can be used to detect signatures of selection. Immune related genes showed the greatest evidence of selection in these families. This underscores the contribution of immune mechanisms and infection susceptibility, to the genetic architecture of severe mental illness.

## Methods

### Study population

The study population consisted of 80 unrelated individuals (Females, N = 34); each of whom was diagnosed with psychiatric illness. The diagnoses were made by two trained psychiatrists based on DSM-IV TR criteria, and included BD (N = 26), schizophrenia (N = 25), dementia (N = 23), OCD (N = 3), SUD (N = 2) and Major Depressive Disorder (N = 1). These individuals were drawn from 80 separate and distinct families who were recruited as a part of the Accelerator Program for Discovery in Brain Disorders using Stem Cells (ADBS) study, which has been approved by the ethics committee of the National Institute of Mental Health and Neurosciences, Bengaluru, India. The study was carried out in accordance with the Declaration of Helsinki for research involving human participants. Written informed consent was obtained from all recruited individuals and their family members, wherever required.

### Sequencing and quality control

As described in our previous study^67^, whole exome sequencing was carried out on the Illumina Hiseq NGS platform with libraries prepared using Illumina exome kits. Reads were aligned with the reference human genome hg19 using the Burrows-Wheeler algorithm tool (https://academic.oup.com/bioinformatics/article/25/14/1754/225615).

### Variant calling

We used bcftools-1.9^68^ to do the variant calling for all our samples from the bam files. First, we used bcftools mpileup to create genotype likelihoods. We then used a minimum base quality of 20 and a minimum mapping quality of 20 to accept it as a true variant. We also used an adjusted mapping quality of 50 to downgrade reads containing excessive mismatches (as recommended in bcftools for BWA). Additionally, we annotated the file using FORMAT/DP so we had depth information in the vcf file. The output was then piped to bcftools call. We used -m for multi allelic caller. We only used SNPs which were present in the gnomAD vcf file using -T command. An example of the code is presented here:

bcftools mpileup–ignore-RG -q 20 -Q 20 -C 50 -r <chr> -a FORMAT/DP-f <ucsc.hg19.fasta>  <*.bam>| bcftools call-m-T <gnomead.vcf.gz> -O z-o <out.vcf.gz>.

A similar approach was also used for the 1000 genome phase 3 release data^69^, where we merged the 1000 genome vcf file using the bcftools merge command. We only kept our target population, Yoruba in Ibadan, Nigeria (YRI), Gujarati Indians in Houston, TX (GIH) and Indian Telugu in the UK (ITU) for further analysis.

### Filtering

After variant calling, we first did a liftover of the vcf file from hg19 to GRCh37 using picard tools. We kept only SNPs (using –remove-indels flag). We removed any genotype where the coverage was less than 10 × using (–minDP 10) and removed any SNPs where we had more than 50% missing genotype data (using –max-missing 0.5). All these commands were done by using vcftools^70^. We kept only unrelated individuals from the affected multiplex families and disease free (control group) individuals for further analysis.

### PBS calculation

We took the vcf file generated from the previous step and used vcftools to create a frequency file for both unrelated individuals and the control group. We used bcftools query to extract information about the frequencies of gnomAD vcf files. We only extracted AF_afr (alternate allele frequency of African-American/African ancestry individuals), AF_sas (alternate allele frequency of South Asian ancestry individuals), AN_afr [(total number of alleles in samples of African-American/African ancestry individuals) and AN_sas (total number of alleles in samples of South Asian ancestry individuals] from the info columns from gnomAD vcf files. These frequency data were used to calculate PBS (X, SAS, AFR) [where X is our data consisting of diseased unrelated individuals] using in house code with scikit-allele^71^. We then extracted the top SNPs by their PBS values and tried to find their impact on phenotype.

We also calculated PBS (X, ITU, YRI) and PBS (X, ITU + GIH, YRI) using scikit-allele to estimate the impact of using a super population instead of using a subpopulation. The R^2^ was calculated using scipy.stat.linregress function from scipy-1.5.3.

### Frequency differentiation between cases and controls

As our target population consists of cases, some of the top PBS values can come from regions which might simply be associated with caseness due to sampling bias. To circumvent this problem, we also calculated the allele frequency differences between case and control data set (|Freq _case_ − Freq _control_|). Subsequently, SNPs (> 99.9th percentile distribution of PBS) were only considered as potential targets of selection if they had allele frequency difference between cases and controls < 99.9th percentile and SNPs with allele frequency difference of > 99.9th percentile of the frequency difference distribution between case and controls were dropped. The rationale being that if the PBS value of a SNP is high due to selection instead of the caseness then the allele frequency difference between cases and controls should not vary as much.

We also implemented an alternative approach (Fisher’s exact test) to find out the top differentiated frequencies in case and control studies. We used plink-1.9.0^72^ –assoc fisher and –allow-no-sex to calculate the p-value and the odds ratio (OR).

### Identification of top candidate genes

We used python to select genes at the top 0.1% (> 99.9th percentile) of the overall PBS distribution and calculated the number of SNPs per gene. We chose 99.9 percentile as significant based on previously published analysis^15^. We thus identified 74 genes as the putative candidates for selection in the target population.

### Analyses of functional enrichment

To perform the enrichment analysis, we used the set of genes obtained via the above method. For GO enrichment, we used the online tool in http://www.geneontology.org/page/go-enrichment-analysis; (GO Ontology database 10.5281/zenodo.5080993 Released 2021-07-02; last accessed August 9, 2021). We analyzed each of the four gene lists with the three GO categories (biological processes, cellular components, and molecular function) using FDR correction with a significance based on P value < 0.05 (ran on 9th August 2021).

The pathway over-representation analysis on the same gene sets was run using the IMPaLA online tool^73^ available at: http://impala.molgen.mpg.de (ran on 8th Dec, 2020) and we considered only pathways with a Q-value less than 0.05 to minimize the false positives because Q-value < 0.05 implies only 5% of results can be false positives.

### Detection of archaic introgression in selected gene regions

We applied *haplostrips* tool to detect archaic introgression^74^ in putative positively selected genes. Haplostrips uses phased genetic data and visualizes polymorphisms of a particular genomic region by clustering and sorting haplotypes independently. The data was phased using shapeit^75^ with 1000 genome third phase reference^69^.

## Supplementary Information

Supplementary Information.

Supplementary Figure S1.

Supplementary Tables.
