# Supplementary material for: Analysis of whole exome sequencing in severe mental illness hints at selection of brain development and immune related genes
Source: Sci Rep. 2021 Oct 26;11:21088. doi: 10.1038/s41598-021-00123-x (PMC8548332; doi:10.1038/s41598-021-00123-x)
Supplement: Supplementary file 1 — Supplementary Information. [file 41598_2021_123_MOESM1_ESM.docx]

**Supplementary data:**

**Table S1:** List of genes with 1 SNP per gene considering SNPs that fall within 99.9^th^ percentile of PBS distribution and are out of 99.9^th^ percentile of difference in the frequency between cases and controls (marked with Yes, otherwise No); the list of SNPs included or excluded is also supported with Fisher’s exact test p-value and odd ratio (OR) to find out the top differentiated frequencies in case and control; the list also contains frequency of each SNP in cases and controls, and also in continental populations extracted from gnomAD.

**Table S2:** List of genes with 2 or more SNPs per gene, considering SNPs that fall within 99.9^th^ percentile of PBS distribution but are out of 99.9^th^ percentile of difference in the frequency between cases and controls (marked with Yes, otherwise No); the list of SNPs included or excluded is also supported with Fisher’s exact test p-value and odd ratio (OR) to find out the top differentiated frequencies in case and control; the list contains frequency of each SNP in cases and controls, and also in continental populations extracted from gnomAD.

**Table S3:** Final list of multiSNP genes (>2 SNP) and single SNP genes with average PBS values after passing the frequency difference cutoffs.

**Table S4:** List of multiSNP genes with plausible association to Schizophrenia, Cognitive ability and Intelligence, and Parkinson’s disease (PD) or Alzheimer’s disease (AD), their description and reference sources.

**Table S5:** GO for enrichment of MultiSNP genes in biological processes.

**Table S6:** GO for enrichment of MultiSNP genes in molecular functions.

**Table S7:** GO for enrichment of MultiSNP genes in Cellular components.

**Table S8:** IMPaLa pathways enrichment analysis results for the list of 74 multiSNP genes altogether.

**Table S9:** List of SNPs (of both the Single SNP and multiSNP genes in the 99.9^th^ percentile of PBS value) that were excluded because they also fall within the 99.9^th^ percentile of the frequency difference between cases and controls, supported with Fisher’s exact test p-value and odd ratio (OR).

**Figure S1:** Relationship between PBS values occurring due to change of reference population between a subpopulation and super population. The x axis is calculated using PBS (case, ITU, YRI) [where case is our target population, ITU is Indian Telugu in the UK and YRI is Yoruba in Ibadan, Nigeria] and y axis is calculated using PBS (case, ITU+GIH, YRI) [where ITU+GIH is Indian Telegu in the UK and Gujarati Indians in Houston, TX together, all the other populations are same as x axis].
