# Supplementary figures and images for: Analysis of whole exome sequencing in severe mental illness hints at selection of brain development and immune related genes
Source: Sci Rep. 2021 Oct 26;11:21088. doi: 10.1038/s41598-021-00123-x (PMC8548332; doi:10.1038/s41598-021-00123-x)

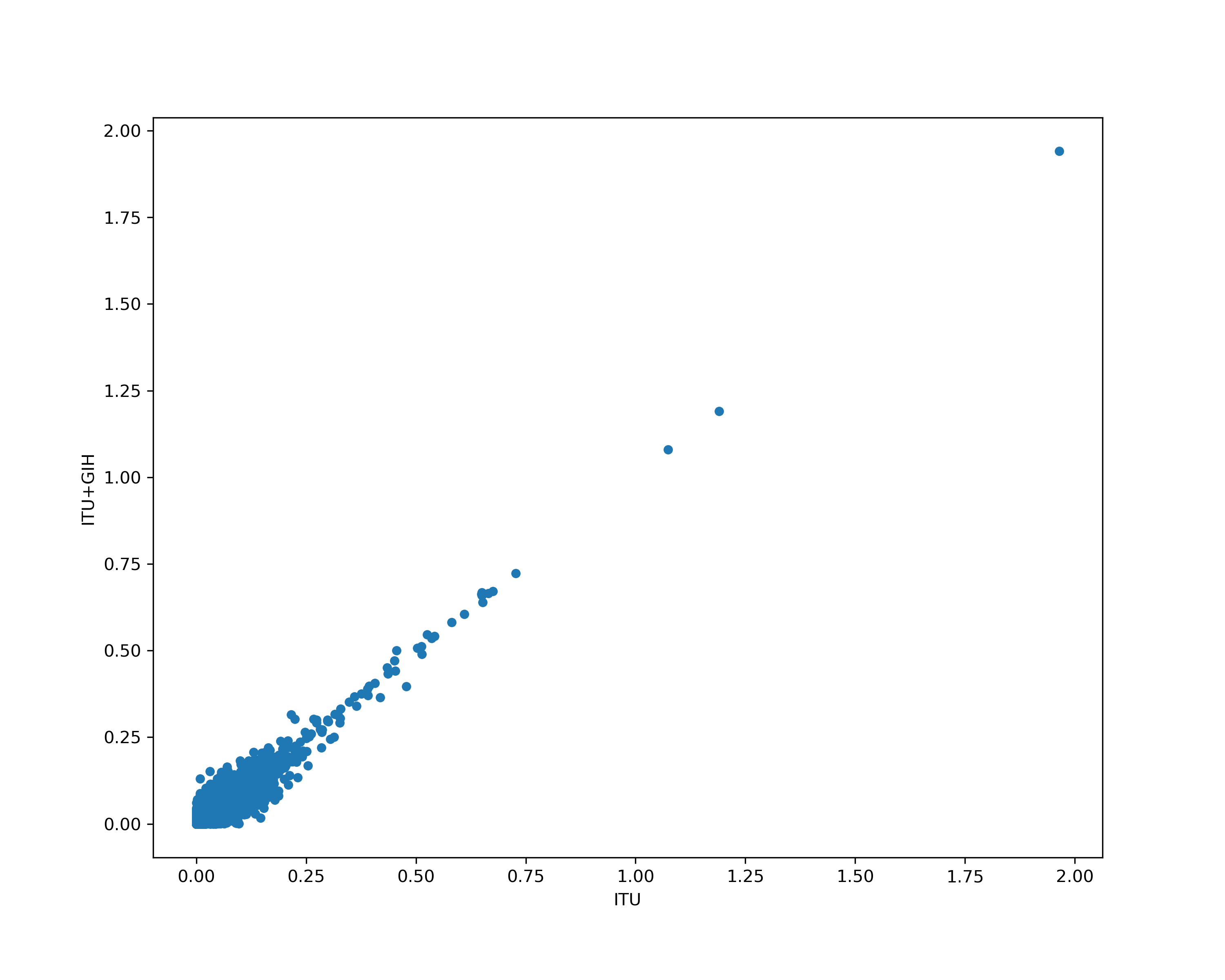

Supplement: Supplementary file 2 — Supplementary Figure S1. [file 41598_2021_123_MOESM2_ESM.png]
